# Cutaneous immune-related adverse events independently predict overall survival with a grade-dependent association in ICI-treated advanced NSCLC: a time-dependent analysis

**DOI:** 10.3389/fonc.2026.1860717

**Published:** 2026-08-12

**Authors:** Tao Xu, Jia Li, Junqi Zhao, Daixi Wu, Zhening Liu, Yumin Zheng, Xinmeng Wang, Ye Zhai, Huijuan Cui

**Affiliations:** 1 Graduate School, Beijing University of Chinese Medicine, Beijing, China; 2 The First Affiliated Hospital of Zhejiang Chinese Medical University (Zhejiang Provincial Hospital of Chinese Medicine), Hangzhou, China; 3 Dongzhimen Hospital, Beijing University of Chinese Medicine, Beijing, China; 4 Department of Integrative Oncology, China-Japan Friendship Hospital, Beijing, China

**Keywords:** cutaneous immune-related adverse events, dose-response, immortal time bias, immune checkpoint inhibitors, landmark analysis, non-small cell lung cancer, overall survival, time-dependent Cox regression

## Abstract

**Introduction:**

Cutaneous immune-related adverse events (cirAE) affect up to 34% of patients receiving immune checkpoint inhibitor (ICI) therapy, yet their prognostic significance in advanced non-small cell lung cancer (NSCLC) remains disputed. A key methodological flaw in the existing literature is immortal time bias, which inflates the apparent benefit of cirAE in conventional analyses.

**Methods:**

We conducted a retrospective cohort study of 262 advanced NSCLC patients treated with ICIs at a single institution between January 2021 and January 2025. Patients were classified into cirAE (n=80) and no cirAE (n=182) groups. Time-dependent Cox regression was the primary analysis to address immortal time bias; landmark analysis at 2.2 months (the median cirAE onset) served as a supportive analysis. The primary endpoint was overall survival (OS); progression-free survival (PFS) was secondary.

**Results:**

In multivariable time-dependent Cox analysis, cirAE was independently associated with improved OS (HR = 0.52; 95% CI: 0.35–0.78; P = 0.002). No time-invariant PFS association was observed (HR = 0.91; P = 0.586); a complementary time-varying analysis revealed an early PFS hazard reduction that attenuated over follow-up, consistent with detection bias from more intensive surveillance in cirAE patients. The OS association was consistent across all prespecified subgroups (all P for interaction >0.05), and was confirmed in a sensitivity analysis re-including patients with concurrent non-cutaneous irAE (HR = 0.56; P = 0.002).An exploratory grade-stratified analysis suggested a severity-associated trend: grade 1–2 (HR = 0.51; P = 0.001) and grade 3+ cirAE (HR = 0.33; P = 0.003) showed progressively lower OS hazard estimates (P for trend <0.001; median OS: 18.8, 36.1, and 47.7 months for no cirAE, grade 1–2, and grade 3+ groups, respectively).

**Discussion:**

Given the small grade 3+ subgroup (n=21), these findings should be considered hypothesis-generating. cirAE may serve as an early, accessible signal of ICI efficacy in NSCLC. The exploratory severity-outcome gradient suggests that high-grade cirAE may identify patients deriving the greatest benefit from continued ICI therapy under appropriate dermatologic management, pending prospective validation.

## 1 Introduction

Lung cancer is the leading cause of cancer death globally, with NSCLC comprising approximately 85% of cases (1). ICIs targeting PD-1, PD-L1, and CTLA-4 have redefined the treatment of advanced NSCLC (2, 3). KEYNOTE-024 and CheckMate-017 established ICI monotherapy as a standard option for PD-L1-high patients (2, 4), while KEYNOTE-189 and KEYNOTE-407 demonstrated that the addition of pembrolizumab to platinum-based chemotherapy significantly improved overall survival in nonsquamous and squamous NSCLC, respectively, regardless of PD-L1 expression (5, 6). Yet durable responses occur in only 20–30% of unselected patients, highlighting the need for better predictive biomarkers (1). PD-L1 expression predicts response imperfectly — approximately 30% of PD-L1-negative patients still respond to ICI — and TMB assessment lacks standardization across platforms (7, 8). Identifying simple, accessible, and early biomarkers of ICI efficacy is therefore a clinical priority.

ICIs exert their antitumor effects by releasing the brakes on T-cell-mediated immunity. This same mechanism, however, can trigger autoimmune-like toxicities collectively termed immune-related adverse events (irAE) (8). irAE and antitumor responses appear to share common immunological pathways: both are driven by T-cell activation against antigens co-expressed on tumor cells and normal tissues (9). Multiple studies report improved survival in ICI-treated patients who develop irAE (10, 11) However, a meta-analysis of 17 studies found that this association was substantially attenuated after correction for immortal time bias (12).A critical methodological concern is immortal time bias: because irAE cannot occur in patients who die early, the pre-event interval is guaranteed “immortal” (13).When irAE status is treated as a fixed baseline variable, the pre-event interval is misclassified as “exposed” person-time, artificially inflating the apparent benefit (14). Studies employing time-dependent analysis to correct for this bias have yielded inconsistent results, with some irAE subtypes losing statistical significance after correction (15).

Among the various irAE subtypes, cirAE deserve special attention. cirAE are the most common irAE subtype, occurring in 24–34% of ICI-treated patients, with a median onset of 4–6 weeks after treatment initiation (16). cirAE differ fundamentally from visceral irAE in manageability. Most cutaneous toxicities—including many grade 3 events—respond to topical corticosteroids and antihistamines, allowing ICI continuation (8, 17). Grade 3+ hepatitis, pneumonitis, or myocarditis, by contrast, typically require high-dose systemic corticosteroids and permanent ICI withdrawal. This distinguishes cirAE from other irAE: higher-grade cutaneous toxicity may signal stronger immune activation while allowing uninterrupted treatment—making cirAE uniquely suited as a prognostic biomarker. Dedicated cirAE studies in NSCLC are scarce and methodologically limited: most have not applied time-dependent analyses to correct for immortal time bias (10, 16). Whether a dose-response relationship exists between cirAE severity and survival remains unclear.

We evaluated the association between cirAE and survival in 262 ICI-treated NSCLC patients, using time-dependent Cox regression and landmark analysis to correct for immortal time bias. We also tested whether OS benefit followed a dose-response gradient across cirAE severity grades.

## 2 Materials and methods

### 2.1 Study design and patient selection

This was a single-center retrospective cohort study conducted at the Department of Oncology, China-Japan Friendship Hospital, Beijing, China. We screened all lung cancer patients who received at least one cycle of ICI therapy between January 2021 and January 2025. The inclusion criteria were: (i) histologically or cytologically confirmed NSCLC; (ii) receipt of at least one ICI agent (anti-PD-1, anti-PD-L1, or anti-CTLA-4); and (iii) clinical stage IIIb or above. The exclusion criteria were: (i) small cell lung cancer (SCLC); (ii) no ICI exposure; (iii) stage earlier than IIIb; (iv) concurrent non-cutaneous irAE (to isolate the independent effect of cirAE); (v) pre-existing autoimmune disease; and (vi) inadequate follow-up (<1 month) or incomplete baseline data.

We excluded patients with concurrent non-cutaneous irAE because these independently predict ICI efficacy and would confound the assessment of cirAE-specific effects. This exclusion affected 36 patients (3.3% of the screened cohort),resulting in a primary analysis cohort of 262 patients. This cohort definition aligns directly with the study’s primary aim to evaluate whether cutaneous toxicity itself carries independent prognostic value. To ensure the robustness of our findings to this exclusion criterion, a sensitivity analysis re-including these 36 patients (total n=298) was performed. The study was approved by the Institutional Ethics Committee of China-Japan Friendship Hospital (approval number:2022-KY-122-2), and the requirement for informed consent was waived given the retrospective design. The study was conducted in accordance with the STROBE guidelines for observational studies (18).

### 2.2 Data collection and definitions

Baseline demographic and clinical data were extracted from the electronic medical record system, including age, sex, Eastern Cooperative Oncology Group performance status (ECOG PS), clinical stage (III, IV), histological type (Non-squamous, Squamous), treatment line (first-line vs. second-line), ICI agent type (anti-PD-1, anti-PD-L1, anti-CTLA-4), and treatment regimen (ICI monotherapy vs. ICI plus chemotherapy).

cirAE were defined as new-onset cutaneous adverse reactions occurring during ICI therapy, confirmed by a dermatologist as ICI-related based on temporal association, morphological pattern, and exclusion of alternative causes. Pre-existing skin conditions were excluded. The severity of cirAE was graded according to the Common Terminology Criteria for Adverse Events (CTCAE) version 5.0 and categorized as grade 1-2 (mild-moderate) or grade 3+ (severe). Based on morphological presentation, cirAE were classified into three types: (i) pruritus/rash (n=42), (ii) inflammatory dermatitis, including eczematous, psoriasiform, lichenoid, and vitiliginous patterns (n=36), and (iii) Stevens-Johnson syndrome/toxic epidermal necrolysis (SJS/TEN; n=2, both grade ≥3). Because of the small number of SJS/TEN cases, these two patients were merged into the inflammatory dermatitis category for descriptive and subgroup analyses, yielding an analytic inflammatory dermatitis category of 38 patients (47.5%); this merged classification is used in **Table 1B** and in all subsequent analyses stratified by morphological type. The time to cirAE onset was defined as the interval from the first ICI dose to the first documented cirAE.

**Table 1A:** Characteristics of cutaneous immune-related adverse events (cirAE) among 80 affected patients.

Characteristic	Value
Incidence of cirAE	80 (30.5%)
Time to cirAE onset, months	
Median (IQR)	2.2 (0.7-9.7)
Range	0.1-49.1
Severity grade	
Grade 1-2	57 (71.2%)
Grade ≥3	23 (28.8%)
Time to onset by grade, median (IQR), months	
Grade 1-2	1.7 (0.5-8.6)
Grade ≥3	3.9 (1.1-10.6)
Type of cirAE	
Pruritus/Rash	42 (52.5%)
Inflammatory dermatitis	38 (47.5%)
Time to onset by type, median (IQR), months	
Pruritus/Rash	1.9 (0.7–5.5)
Inflammatory dermatitis	2.6 (0.5–12.8)
Severity by type, n (%)	
Pruritus/Rash, Grade 1–2	32 (40.0%)
Pruritus/Rash, Grade ≥3	10 (12.5%)
Inflammatory dermatitis, Grade 1–2	25 (31.2%)
Inflammatory dermatitis, Grade ≥3	13 (16.2%)

Data are presented as n (%) or median (IQR).Inflammatory dermatitis includes eczematous dermatitis, lichenoid dermatitis, vitiliginous changes, psoriasiform, and Stevens–Johnson syndrome/toxic epidermal necrolysis (SJS/TEN; n = 2, both Grade ≥3), merged into this category for analysis due to the small number of SJS/TEN cases (see Methods). Severity grading was performed according to Common Terminology Criteria for Adverse Events (CTCAE) version 5.0. cirAE, cutaneous immune-related adverse event; IQR, interquartile range.

**Table 1B:** Management of cutaneous immune-related adverse events and impact on ICI continuation, stratified by severity grade.

Treatment	Grade 1-2 (n=57)	Grade 3+ excl. SJS/TEN (n=21)	SJS/TEN (n=2)
Topical corticosteroids	51 (89.5%)	20 (95.2%)	2 (100%)
Oral antihistamines	43 (75.4%)	19 (90.5%)	1 (50.0%)
Emollients	48 (84.2%)	17 (81.0%)	0 (0%)
Systemic corticosteroids	3 (5.3%)	16 (76.2%)	2 (100%)
IVIG or other immunosuppressants	0 (0%)	0 (0%)	1 (50.0%)
ICI treatment impact			
ICI continued without interruption	54 (94.7%)	9 (42.9%)	0 (0%)
ICI temporarily held	3 (5.3%)	12 (57.1%)	0 (0%)
Duration, median (IQR), days	7 (5–10)	18(10–24)	28 (21–35)
Duration of hold, median (IQR), days	14 (10–21)	21 (14–28)	—
Successfully resumed ICI	3/3 (100%)	11/12 (91.7%)	—
ICI permanently discontinued	0 (0%)	1 (4.8%)	2 (100%)
Overall ICI continuation rate	57/57 (100%)	20/21 (95.2%)	0/2 (0%)

**Table 1B** Management was conducted in accordance with the ASCO (17)and ESMO (23, 25) clinical practice guidelines for immune-related adverse events. ICI continuation rate is defined as the proportion of patients who either continued ICI without interruption or successfully resumed ICI after a temporary hold. IVIG, intravenous immunoglobulin; IQR, interquartile range.

The primary endpoint was overall survival, defined as the time from the first ICI dose to death from any cause or last follow-up. The secondary endpoint was progression-free survival, defined as the time from the first ICI dose to disease progression per RECIST v1.1 or death. The data cutoff date was March 1, 2026.

### 2.3 Statistical analysis

Baseline characteristics were summarized as counts and percentages for categorical variables and as medians with interquartile ranges (IQR) for continuous variables. Between-group comparisons used the chi-square test or Fisher’s exact test for categorical variables and the Wilcoxon rank-sum test for continuous variables. All tests were two-sided, with P < 0.05 considered statistically significant.

A central methodological challenge in irAE-survival studies is immortal time bias (13, 14). Because cirAE occurrence requires a period of ICI exposure, patients in the cirAE group are guaranteed to survive at least until the event occurs. Treating cirAE as a fixed covariate in conventional Cox regression misclassifies this “immortal” period as exposed person-time, leading to overestimation of the protective effect. To address this bias, we employed two complementary approaches.

First, we performed landmark analysis. The landmark time point was set at 2.2 months, corresponding to the median time to cirAE onset in our cohort. Patients who died or were lost to follow-up before the landmark were excluded, and survival was measured from the landmark time point onward (19). Patient cirAE status was determined at the landmark: patients with cirAE onset before or at 2.2 months were classified as the cirAE group; all remaining patients—including those who subsequently developed cirAE after the landmark—were classified as the no cirAE group. We acknowledge an important practical limitation of this approach in our cohort: because all 262 patients survived beyond the 2.2-month landmark, no patient was ultimately excluded by the landmark requirement. Consequently, the landmark analysis as implemented here functions as a delayed-entry comparison rather than a strict exclusion-based sensitivity analysis. It is therefore best regarded as a complementary visualization of the time-dependent Cox findings rather than as an independent correction for immortal time bias. Kaplan-Meier curves with log-rank tests were generated from the landmark (**Figures 2C, D**, **3**).

Second, we used time-dependent Cox proportional hazards regression, in which cirAE was modeled asa time-varying covariate (20). Under this framework, each patient contributed person-time to the “unexposed” state from ICI initiation until cirAE onset, and to the “exposed” state thereafter. Patients who never developed cirAE remained in the unexposed state throughout their follow-up. This approach avoids the misattribution of pre-event survival time to the cirAE group. TD Cox was used for all regression analyses, including univariable screening, multivariable adjustment, subgroup interaction testing, and grade-stratified comparisons (**Tables 2**–**4**).

**Table 2A:** Univariable and multivariable time-dependent Cox proportional hazards regression analysis for overall survival.

Variable	Univariable HR (95% CI)	P value	Multivariable HR (95% CI)	P value
cirAE†	0.48 (0.33 to 0.69)	< 0.001	0.52 (0.35 to 0.78)	0.002
Sex				
Female	1 [Reference]		1 [Reference]	
Male	0.84 (0.57 to 1.23)	0.375	0.97 (0.64 to 1.46)	0.876
Age, years				
< 65	1 [Reference]		1 [Reference]	
≥ 65	0.93 (0.68 to 1.27)	0.639	0.82 (0.59 to 1.13)	0.227
ECOG PS				
0–1	1 [Reference]		1 [Reference]	
2	1.20 (0.61 to 2.38)	0.601	1.31 (0.62 to 2.78)	0.475
Clinical stage				
III	1 [Reference]		1 [Reference]	
IV	3.16 (2.05 to 4.87)	< 0.001	3.16 (1.99 to 5.01)	< 0.001
Histology				
Non-squamous	1 [Reference]		1 [Reference]	
Squamous	0.96 (0.71 to 1.30)	0.791	1.34 (0.97 to 1.87)	0.080
PD-L1 TPS				
< 1%	1 [Reference]		1 [Reference]	
1–49%	0.56 (0.37 to 0.83)	0.004	0.44 (0.29 to 0.67)	< 0.001
≥ 50%	0.60 (0.36 to 1.00)	0.050	0.37 (0.22 to 0.63)	< 0.001
Unknown	1.10 (0.75 to 1.62)	0.635	0.99 (0.66 to 1.47)	0.951
Treatment regimen				
Chemotherapy + ICI	1 [Reference]		1 [Reference]	
ICI monotherapy	1.39 (0.94 to 2.04)	0.096	1.66 (0.77 to 3.55)	0.196
Line of therapy				
First-line	1 [Reference]		1 [Reference]	
Second-line	1.13 (0.79 to 1.61)	0.51	0.69 (0.35 to 1.38)	0.293
C-statistic			0.676	

Hazard ratios were estimated using time-dependent Cox proportional hazards regression. cirAE was modeled as a time-dependent covariate. All pre-specified clinical variables were included in the multivariable model regardless of univariable significance, as the number of outcome events was sufficient to support full model adjustment (174 events for OS; 218 events for PFS). All variance inflation factors were < 5. CI, confidence interval; cirAE, cutaneous immune-related adverse event; HR, hazard ratio; OS, overall survival.

**Table 2B:** Univariable and Multivariable time-dependent Cox proportional hazards regression analysis for progression-free survival.

Variable	Univariable HR (95% CI)	P value	Multivariable HR (95% CI)	P value
cirAE†	0.73 (0.54 to 1.00)	0.05	0.91 (0.65 to 1.28)	0.586
Sex				
Female	1 [Reference]		1 [Reference]	
Male	0.74 (0.53 to 1.02)	0.069	0.84 (0.59 to 1.20)	0.337
Age, years				
< 65	1 [Reference]		1 [Reference]	
≥ 65	0.95 (0.72 to 1.25)	0.707	0.86 (0.64 to 1.15)	0.309
ECOG PS				
0–1	1 [Reference]		1 [Reference]	
2	1.77 (0.96 to 3.25)	0.066	2.31 (1.18 to 4.51)	0.014
Clinical stage				
III	1 [Reference]		1 [Reference]	
IV	2.29 (1.63 to 3.22)	< 0.001	2.60 (1.78 to 3.78)	< 0.001
Histology				
Non-squamous	1 [Reference]		1 [Reference]	
Squamous	1.03 (0.79 to 1.35)	0.802	1.49 (1.11 to 2.00)	0.009
PD-L1 TPS				
< 1%	1 [Reference]		1 [Reference]	
1–49%	0.63 (0.44 to 0.89)	0.009	0.57 (0.40 to 0.82)	0.002
≥ 50%	0.67 (0.43 to 1.05)	0.083	0.43 (0.27 to 0.70)	< 0.001
Unknown	0.95 (0.67 to 1.36)	0.79	0.89 (0.62 to 1.28)	0.53
Treatment regimen				
Chemotherapy + ICI	1 [Reference]		1 [Reference]	
ICI monotherapy	1.56 (1.12 to 2.19)	0.009	2.20 (1.23 to 3.91)	0.008
Line of therapy				
First-line	1 [Reference]		1 [Reference]	
Second-line	1.27 (0.93 to 1.74)	0.134	0.61 (0.36 to 1.05)	0.074
C-statistic			0.642	

**Tables 3B** Same methodology as **Table 2A**, with PFS as the endpoint. PFS, progression-free survival.

**Table 3:** Subgroup analysis of the association between cutaneous immune-related adverse events and overall survival.

Subgroup	No. of patients	HR (95% CI)	P for interaction
Overall	262	0.48 (0.33-0.69)	
Sex			
Female	51	0.25 (0.08-0.84)	0.335
Male	211	0.52 (0.35-0.77)	
Age			
< 65 years	94	0.28 (0.13-0.57)	0.057
≥ 65 years	168	0.61 (0.40-0.93)	
ECOG PS			
0-1	250	0.48 (0.33-0.69)	0.936
2	12	0.64 (0.13-3.25)	
Clinical stage			
III	65	0.30 (0.13-0.74)	0.160
IV	197	0.68 (0.46-1.02)	
Histology			
Non-squamous	122	0.48 (0.28-0.80)	0.927
Squamous	140	0.47 (0.28-0.79)	
PD-L1 TPS			
< 1%	71	0.33 (0.17-0.64)	0.149
1-49%	84	0.57 (0.29-1.16)	
≥50%	36	0.77 (0.26-2.30)	
Treatment regimen			
ICI monotherapy	48	0.41 (0.10-1.74)	0.664
Chemotherapy + ICI	214	0.49 (0.33-0.72)	
Line of therapy			
First-line	203	0.44 (0.29-0.66)	0.511
Second-line	59	0.69 (0.32-1.47)	

cirAE, cutaneous immune-related adverse event; ECOG PS, Eastern Cooperative Oncology Group performance status; CI, confidence interval; HR, hazard ratio; ICI, immune checkpoint inhibitor; PD-L1, programmed death-ligand 1; TPS, tumor proportion score. Hazard ratios represent the effect of cirAE as a time-dependent covariate on overall survival within each subgroup. P for interaction was calculated using a likelihood ratio test comparing models with and without the cirAE-by-subgroup interaction term.

**Table 4:** Association between cirAE severity and overall survival.

Group	n	Events (%)	Median OS, months (95% CI)	HR (95% CI)	P value
No cirAE	182	135 (74.2)	18.8 (16.9–22.4)	Reference	—
G1–2 cirAE	57	29 (50.9)	36.1 (31.0–NR)	0.51 (0.34–0.76)	0.001
G3+ cirAE	21	8 (38.1)	47.7 (33.5–NR)	0.33 (0.16–0.69)	0.003
P for trend					< 0.001

Two patients with SJS/TEN were excluded (n = 260). Hazard ratios were estimated using time-dependent Cox regression with no cirAE as the reference. P for trend was assessed by modeling cirAE grade as an ordinal variable (0/1/2); HR per grade increase = 0.54 (95% CI: 0.41-0.73). CI, confidence interval; HR, hazard ratio; NR, not reached; OS, overall survival; SJS/TEN, Stevens-Johnson syndrome/toxic epidermal necrolysis.

Landmark analysis provided visual comparisons; TD Cox enabled regression adjustment. Concordance between the two methods would support the robustness of our conclusions. Consistency between the two approaches would increase confidence in the validity of the observed associations.

For multivariable analysis, all prespecified baseline clinical variables (including age, sex,ECOG PS, clinical stage, histology, PD-L1 TPS, treatment regimen, and line of therapy) were included in the time-dependent Cox model alongside cirAE status, regardless of their univariable significance, to comprehensively adjust for potential confounding. Multicollinearity was assessed using variance inflation factors (VIF); a VIF > 5 was considered indicative of problematic collinearity. Subgroup analyses were performed for prespecified clinical variables. The interaction between cirAE status and each subgroup variable was tested by including a product term in the TD Cox model; P for interaction < 0.05 was considered indicative of effect modification. Results were displayed as a forest plot. Proportional-hazards assumptions were assessed using scaled Schoenfeld residuals. Variables violating the assumption were handled by stratification for covariates or by time-varying coefficient models for the exposure of interest. Three patients in the cirAE group experienced PFS events before cirAE onset and were classified as unexposed for PFS analysis. Total ICI cycles and treatment duration are reported in **Table 5** but were not modeled as covariates. These metrics are determined by survival itself and lie on the causal pathway from cirAE to OS rather than acting as baseline confounders. Adjusting for them would induce reverse-causation bias.

**Table 5:** Baseline demographic and clinical characteristics of the study cohort according to cirAE status.

Characteristic	Overall	cirAE	No cirAE	P value
	N = 262	n = 80	n = 182	
Sex				0.131
Male	211 (80.5%)	69 (86.2%)	142 (78.0%)	
Female	51 (19.5%)	11 (13.8%)	40 (22.0%)	
Age				0.330
< 65 years	94 (35.9%)	25 (31.2%)	69 (37.9%)	
≥65 years	168 (64.1%)	55 (68.8%)	113 (62.1%)	
ECOG				0.760
0-1	250 (95.4%)	76 (95.0%)	174 (95.6%)	
2	12 (4.6%)	4 (5.0%)	8 (4.4%)	
Clinical stage				< 0.001
III	65 (24.8%)	35 (43.8%)	30 (16.5%)	
IV	197 (75.2%)	45 (56.2%)	152 (83.5%)	
Histology				0.687
Non-squamous	122 (46.6%)	39 (48.8%)	83 (45.6%)	
Squamous	140 (53.4%)	41 (51.2%)	99 (54.4%)	
PD-L1 TPS				0.726
< 1%	71 (27.1%)	25 (31.2%)	46 (25.3%)	
1-49%	84 (32.1%)	26 (32.5%)	58 (31.9%)	
≥50%	36 (13.7%)	9 (11.2%)	27 (14.8%)	
Unknown	71 (27.1%)	20 (25.0%)	51 (28.0%)	
Treatment regimen				< 0.001
Chemotherapy + ICI	214 (81.7%)	75 (93.8%)	139 (76.4%)	
ICI monotherapy	48 (18.3%)	5 (6.2%)	43 (23.6%)	
Line of therapy				0.630
First-line	203 (77.5%)	64 (80.0%)	139 (76.4%)	
Second-line	59 (22.5%)	16 (20.0%)	43 (23.6%)	
Median follow-up, months (95% CI)	34.2 (32.4–40.0	37.1 (33.1–46.3)	33.5 (30.1–46.4)	–
Median number of ICI cycles (IQR)	7.0 (4.0–15.8)	14.0 (6.0–24.0)	6.0 (4.0–12.0)	< 0.001
Median ICI treatment duration, months (IQR)	5.4 (3.0–12.0)	9.6 (4.3–17.2)	4.8 (2.9–8.8)	< 0.001

Data are presented as n (%). P values were calculated using Fisher’s exact test with 10,000 Monte Carlo simulations. cirAE, cutaneous immune-related adverse event; ECOG PS, Eastern Cooperative Oncology Group performance status; ICI, immune checkpoint inhibitor; PD-L1, programmed death-ligand 1; TPS, tumor proportion score. Median follow-up was estimated by the reverse Kaplan–Meier method.

To explore the dose-response relationship between cirAE severity and OS, patients were stratified into three groups: no cirAE, grade 1–2 cirAE, and grade 3+ cirAE. Two patients with SJS/TEN were excluded from the grade-stratified analysis. SJS/TEN is pathophysiologically distinct from other cirAE: it is driven by drug hypersensitivity rather than T-cell-mediated immunity, carries a mortality rate of 10–30%, and mandates permanent ICI discontinuation (21, 22). Including SJS/TEN would confound the dose-response analysis, as its prognosis is driven by toxicity rather than immune efficacy (22).P for trend was calculated by entering cirAE grade as an ordinal variable (0 = no cirAE, 1 = grade 1-2, 2 = grade 3+) in a TD Cox model. All analyses were performed using R version 4.4.1 (R Foundation for Statistical Computing, Vienna, Austria). Key packages included survival, survminer, forestploter, and ggplot2.

## 3 Results

### 3.1 Patient characteristics

**Figure 1:**
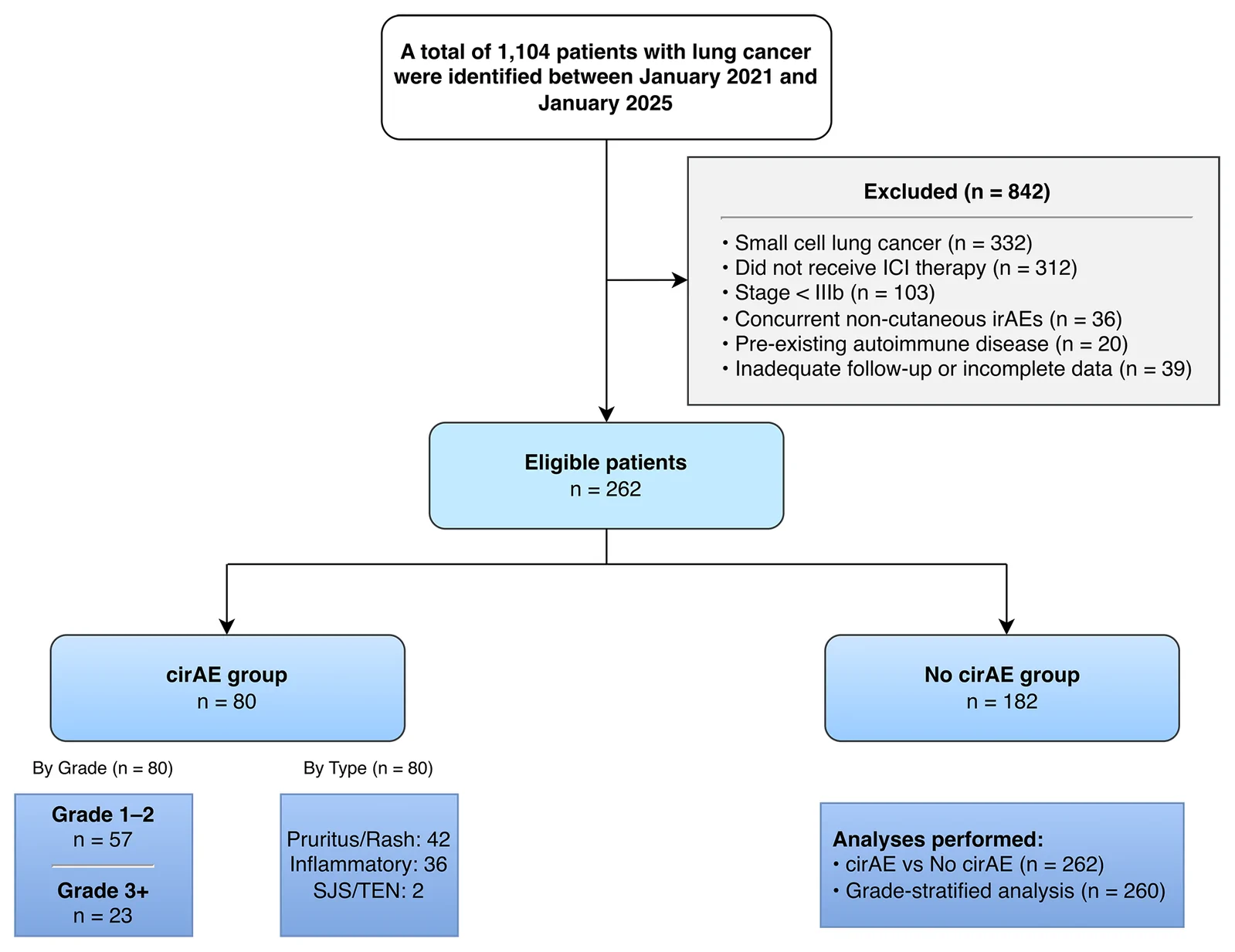
Study flow diagram. A total of 1,104 lung cancer patients were screened. After applying the exclusion criteria, 262 patients were included and classified into the cirAE group (n = 80) and the no cirAE group (n = 182). The cirAE group was further categorized by severity grade (grade 1–2 vs. grade 3+) and morphological type (pruritus/rash, inflammatory dermatitis, SJS/TEN). cirAE, cutaneous immune-related adverse event; ICI, immune checkpoint inhibitor; irAE, immune-related adverse event; NSCLC, non-small cell lung cancer; SCLC, small cell lung cancer; SJS/TEN, Stevens-Johnson syndrome/toxic epidermal necrolysis.

A total of 1,104 lung cancer patients were screened, and 262 met the eligibility criteria (**Figure 1**). The sequential exclusions were as follows: SCLC (n = 332), no ICI treatment (n = 312), stage below IIIb (n = 103), concurrent non-cutaneous irAE (n = 36), pre-existing autoimmune disease (n = 20), and insufficient follow-up or incomplete data (n = 39). Of the 262 included patients, 80 (30.5%) developed cirAE during ICI therapy, while 182 (69.5%) did not.

Baseline characteristics of the two groups are summarized in **Table 5**. The majority of patients were aged≥65 years (64.1%), and 80.5% were male. The two groups were well matched for most baseline characteristics, except for clinical stage (P<0.001) and treatment regimen (P<0.001), with the cirAE group comprising a higher proportion of stage III patients (43.8% vs. 16.5%) and more receiving chemo-ICI combinations (93.8% vs. 76.4%). These imbalanced variables were included as covariates in the multivariable model. The median follow-up of the cohort was 34.2 months (95% CI: 32.4–40.0), estimated by the reverse Kaplan–Meier method. Median follow-up was comparable between the cirAE and no cirAE groups (37.1 vs 33.5 months), whereas patients in the cirAE group received a substantially greater number of ICI cycles (median 14.0 vs 6.0; P < 0.001) and longer total ICI treatment duration (median 9.6 vs 4.8 months; P < 0.001). The methodological treatment of these treatment-exposure variables is detailed in the Methods and Limitations.

### 3.2 Characteristics of cutaneous immune-related adverse events

The clinical characteristics of cirAE are detailed in **Table 1A**. The median time to cirAE onset was 2.2 months (IQR: 0.7-9.7). The most common morphological type was pruritus/rash (n = 42, 52.5%), followed by inflammatory dermatitis (n = 36, 45.0%) and SJS/TEN (n = 2, 2.5%). Regarding severity, 57 patients (71.2%) had grade 1–2 events and 23 (28.8%) had grade 3 or higher, including the two SJS/TEN cases. The management strategies for cirAE and their impact on ICI continuation are summarized in **Table 1A**. All cirAE were managed in accordance with the ASCO and ESMO clinical practice guidelines for immune-related adverse events (17, 23).

Among the 57 patients with grade 1–2 cirAE, topical corticosteroids were the mainstay of treatment (n = 51, 89.5%), frequently combined with oral antihistamines (n = 43, 75.4%) and emollients (n = 48, 84.2%). Systemic corticosteroids were required in only 3 patients (5.3%), all at low doses (prednisone ≤0.5 mg/kg/day) for a short duration (median 7 days). ICI therapy was temporarily held in 3 patients (5.3%) for a median of 14 days (IQR: 10–21), all of whom successfully resumed treatment after symptom improvement. No patient with grade 1–2 cirAE required permanent ICI discontinuation. The overall ICI continuation rate in this group was 100%.

Among the 21 patients with grade 3+ cirAE excluding SJS/TEN, topical corticosteroids were used in 20 patients (95.2%) and oral antihistamines in 19 (90.5%). Systemic corticosteroids were administered in 16 patients (76.2%), at doses of prednisone 0.5–1.0 mg/kg/day, with a median treatment duration of 18 days (IQR: 10–24). ICI therapy was temporarily held in 12 patients (57.1%) for a median of 21 days (IQR: 14–28). Of these 12, 11 (91.7%) successfully resumed ICI after dermatologic reassessment, with no recurrence of grade 3+ toxicity in 8 cases and manageable recurrence at a lower grade in 3 cases. Only one patient (4.8%) required permanent ICI discontinuation. The overall ICI continuation rate (defined as continued ICI therapy without interruption or resumed ICI after temporary hold) in the grade 3+ non-SJS/TEN group was 95.2% (20/21).

The two patients with SJS/TEN were managed with high-dose intravenous methylprednisolone (2 mg/kg/day), and one additionally received intravenous immunoglobulin (IVIG). Both requiredpermanent ICI discontinuation as mandated by current guidelines. Both patients survived the acute episode but did not resume immunotherapy.

A summary of management outcomes by cirAE grade is presented below:

### 3.3 Association between cirAE and overall survival

**Figure 2:**
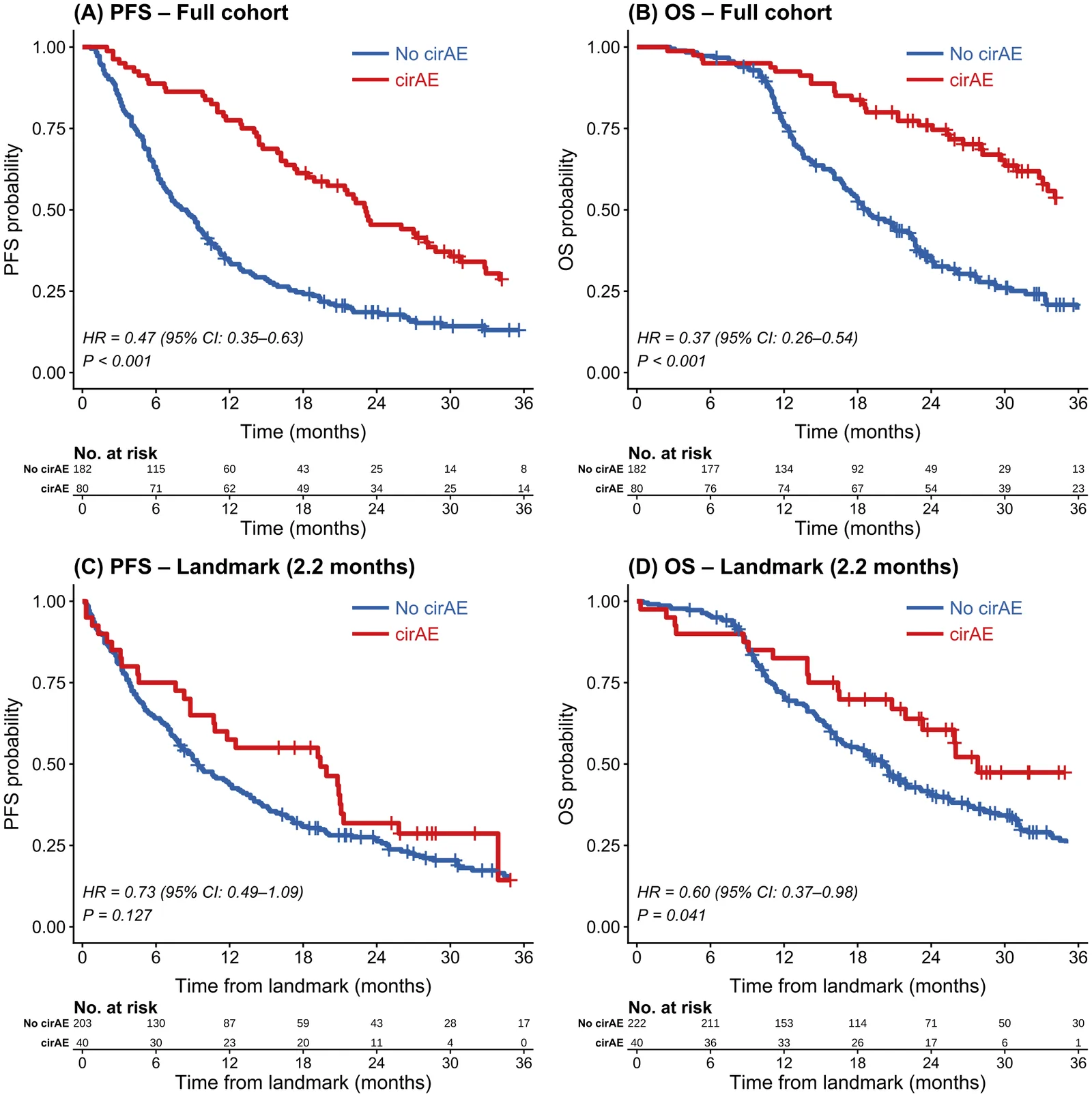
Kaplan-Meier survival curves for overall survival (OS) and progression-free survival (PFS) according to cirAE status. **(A)** PFS in the full cohort. **(B)** OS in the full cohort. **(C)** PFS using landmark analysis at 2.2 months. **(D)** OS using landmark analysis at 2.2 months (the landmark time represents the median time to cirAE onset). P values were calculated using the log-rank test. Hazard ratios (HRs) and 95% confidence intervals (CIs) for these visualizations were determined using unadjusted conventional Cox regression **(A, B)** and landmark Cox regression **(C, D)** to illustrate the impact of immortal time bias prior to time-dependent modeling. Blue lines indicate patients without cirAEs; red lines indicate patients with cirAEs.

Unadjusted Kaplan-Meier analysis showed significantly longer OS in the cirAE group (**Figure 2B**; log-rank P < 0.001). However, this comparison is susceptible to immortal time bias. After landmark adjustment at 2.2 months, the survival advantage persisted (**Figure 2D**; log-rank P = 0.041). Notably, all 262 patients had an OS exceeding 2.2 months; therefore, no patients were excluded by the landmark. The median OS was 36.7 months (95% CI: 32.8–not reached) in the cirAE group versus 18.8 months (95% CI: 16.9–22.4) in the no cirAE group.”

**Figure 3:**
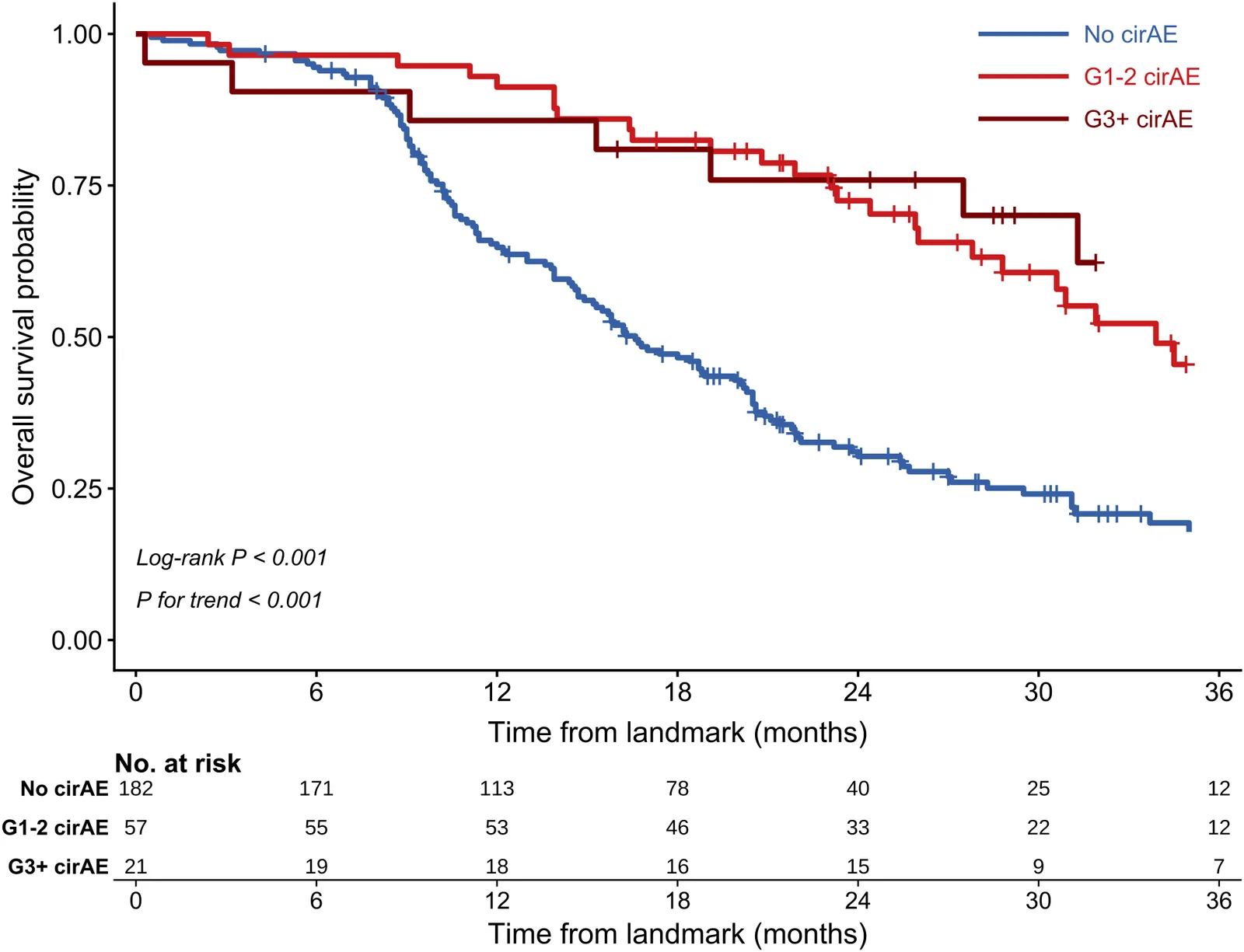
Kaplan-Meier curves for overall survival stratified by cirAE severity grade. Patients were classified into no cirAE (n = 182; blue), grade 1–2 cirAE (n = 57; red), and grade 3+ cirAE (n = 21; dark red). Two patients with Stevens-Johnson syndrome/toxic epidermal necrolysis were excluded from this analysis (see Methods). Landmark analysis was performed at 2.2 months. P for trend was calculated by entering cirAE grade as an ordinal variable (0 = no cirAE, 1 = grade 1-2, 2 = grade 3+) in a time-dependent Cox model; HR per grade increase = 0.54 (95% CI: 0.41-0.73). CI, confidence interval; HR, hazard ratio.

Insert **Figure 2** here - KM curves for OS and PFS.

In univariable TD Cox analysis, cirAE was significantly associated with a reduced risk of death (HR = 0.48; 95% CI: 0.33-0.69; P < 0.001). After multivariable adjustment for all prespecified baseline characteristics, cirAE remained an independent predictor of improved OS (HR = 0.52; 95% CI: 0.35-0.78; P = 0.002) (**Table 2A**). Other independent prognostic factors in the multivariable model included clinical stage and PD-L1 TPS. VIF values for all covariates were below 5, confirming the absence of multicollinearity.

### 3.4 Association between cirAE and progression-free survival

In contrast to the OS findings, cirAE showed no true independent association with PFS. While unadjusted Kaplan-Meier analysis suggested a significantly longer PFS in the cirAE group (**Figure 2A**; log-rank P < 0.001), this apparent benefit was eliminated after landmark adjustment (**Figure 2C**; log-rank P = 0.127), highlighting the profound impact of immortal time bias. This lack of association was confirmed by TD Cox regression: in the multivariable model, cirAE was not independently associated with PFS (HR = 0.91; 95% CI: 0.65-1.28; P = 0.586) (**Table 2B**). The discrepancy between OS and PFS findings is addressed in the Discussion.

### 3.5 Subgroup analysis

**Figure 4:**
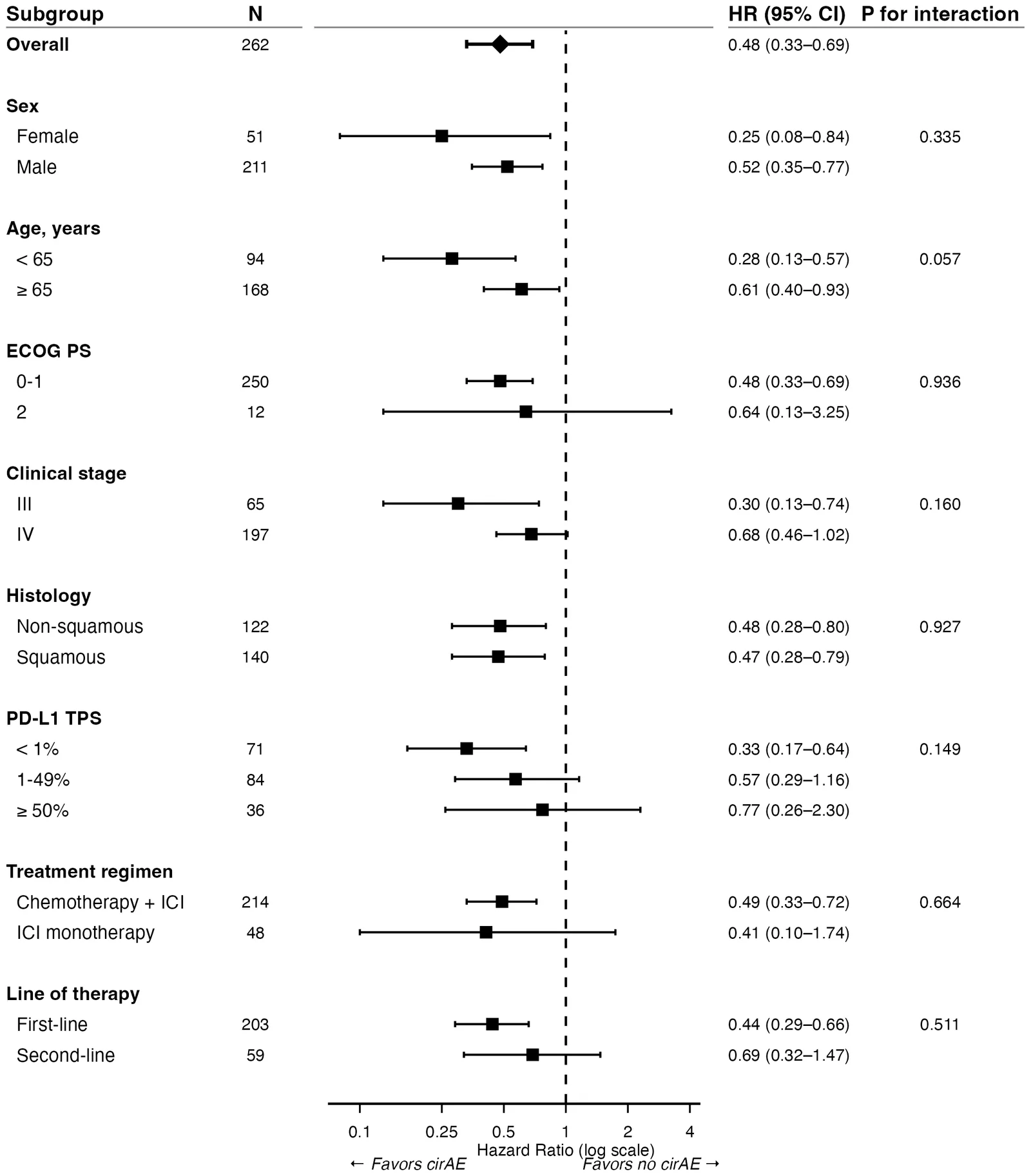
Forest plot of subgroup analyses for the association between cirAE and overall survival. Hazard ratios (HRs) and 95% confidence intervals (CIs) were estimated using time-dependent Cox proportional hazards regression. P for interaction was calculated by including the interaction term between cirAE status (as a time-varying covariate) and each subgroup variable in the model. The vertical dashed line indicates HR = 1.0 (null effect). Values to the left of the dashed line (Favors cirAE) indicate a reduced risk of death associated with the occurrence of cirAEs. CI, confidence interval; ECOG PS, Eastern Cooperative Oncology Group performance status; HR, hazard ratio; ICI, immune checkpoint inhibitor; PD-L1, programmed death-ligand 1; TPS, tumor proportion score.

Subgroup interaction analyses confirmed the consistency of the cirAE-OS association across all prespecified clinical variables (**Figure 4**; **Table 3**). HR point estimates favored cirAE (HR < 1.0) in all subgroups, including those defined by age, sex, ECOG PS, clinical stage, histology, treatment line, and regimen. None of the interaction terms reached statistical significance (all P for interaction > 0.05), indicating that the protective effect of cirAE on OS was consistent and not significantly modified by any of the clinical characteristics assessed. Although the 95% confidence intervals crossed 1.0 in certain subgroups (ECOG PS 2, clinical stage IV, PD-L1 TPS 1-49%, PD-L1 TPS ≥ 50%, ICI monotherapy, and second-line therapy), this was likely attributable to the limited sample sizes within these specific strata rather than a true lack of effect.

### 3.6 Grade-stratified analysis: dose-response relationship

After excluding two patients with SJS/TEN (see Methods for rationale), 260 patients were included in the grade-stratified analysis: no cirAE (n = 182), grade 1–2 cirAE (n = 57), and grade 3+ cirAE (n = 21).

Landmark Kaplan-Meier analysis revealed a clear separation of the three survival curves (**Figure 3**; log-rank P < 0.001). The median OS increased stepwise with cirAE severity: 18.8 months (95% CI: 16.9-22.4) for no cirAE, 36.1 months (95% CI: 31.0-not reached) for grade 1-2, and 47.7 months (95% CI: 33.5-not reached) for grade 3+ cirAE.

TD Cox regression confirmed these findings (**Table 1B**). Using no cirAE as the reference, grade 1–2 cirAE was associated with a 49% reduction in the risk of death (HR = 0.51; 95% CI: 0.34-0.76; P = 0.001), while grade 3+ cirAE was associated with a 67% reduction (HR = 0.33; 95% CI: 0.16-0.69; P = 0.003). The lower HR in the grade 3+ group compared to grade 1–2 suggests a dose-response relationship, which was formally confirmed by the trend test: the HR per grade increase was 0.54 (95% CI: 0.41-0.73; P for trend < 0.001). The event rate was lowest in the grade 3+ group (38.1%), compared with 50.9% for grade 1–2 and 74.2% for no cirAE.

### 3.7 Proportional-hazards testing and time-varying coefficient analysis

To verify the Cox proportional-hazards (PH) framework, we assessed the PH assumption using scaled Schoenfeld residuals. For OS, the cirAE term satisfied the PH assumption (P = 0.820), supporting the time-dependent Cox-derived adjusted HR of 0.52. ECOG performance status showed a mild PH violation (P = 0.028); a sensitivity analysis stratifying on ECOG yielded a materially identical cirAE estimate (HR = 0.51; 95% CI: 0.34–0.77) and resolved the violation (global P = 0.58).

For PFS, the cirAE term violated the PH assumption (P = 0.013). We therefore fitted a complementary time-varying coefficient model parameterized as *log HR(t) = β1 × cirAE + β2 × [cirAE × log(t)]*. The cirAE × log(t) interaction was statistically significant (*β2* = +0.577; 95% CI: +0.102 to +1.053; P = 0.017), alongside a strongly negative main effect (*β1* = -1.542; 95% CI: -2.81 to -0.27; P = 0.018). This combination indicates that cirAE was associated with a substantial early reduction in progression hazard that attenuated monotonically over follow-up. Other covariates generally satisfied the PH assumption; stratifying on those with borderline departures did not meaningfully alter the cirAE coefficient estimates.

### 3.8 Sensitivity analysis re-including patients with concurrent non-cutaneous irAE

To assess whether the exclusion of patients with concurrent non-cutaneous irAE affected the primary conclusions, we performed a sensitivity analysis re-including these 36 patients in an expanded cohort of 298 patients (116 with any cirAE, including 80 with isolated cirAE and 36 with concurrent non-cutaneous irAE; 182 with no irAE of any kind). All 36 re-included patients had cirAE that was concurrent with at least one non-cutaneous irAE; the most common concurrent non-cutaneous irAEs were thyroiditis, hepatitis, and pneumonitis. The median follow-up of the expanded cohort and the distribution of baseline characteristics did not differ substantively from the primary cohort.

Time-dependent Cox regression in the expanded cohort yielded results consistent in direction and magnitude with the primary analysis (**Supplementary Tables 1A, B**). After multivariable adjustment for all baseline covariates, the presence of any cirAE remained independently associated with improved OS [HR = 0.56; 95% CI: 0.39-0.81; P = 0.002] and was not associated with PFS [HR = 1.00; 95% CI: 0.73-1.37; P = 0.989]. The point estimate for the OS hazard ratio in the expanded cohort was similar to that in the primary cohort (HR = 0.52), and the direction and statistical significance of all other covariates were preserved. These findings indicate that the primary results are robust to the inclusion or exclusion of patients with concurrent non-cutaneous irAE, supporting the validity of cirAE as an independent prognostic signal in ICI-treated advanced NSCLC.

## 4 Discussion

Three findings emerged from this analysis. First, cirAE was independently associated with improved OS (HR = 0.52) after correction for immortal time bias—a methodological step absent in most prior studies. Second, this protective association was absent for PFS in conventional time-invariant analyses; a time-varying coefficient analysis clarified that cirAE was associated with a strong early reduction in progression hazard that attenuated over follow-up, reconciling the apparent OS–PFS discordance with a single underlying signal. Third, an exploratory grade-stratified analysis suggested a severity-associated trend: grade 3+ cirAE was associated with the largest point estimate of OS benefit (HR = 0.33; median OS 47.7 months), although the limited size of this subgroup warrants cautious interpretation. These findings carry both biological and clinical implications, which we examine below.

Our results align with a growing body of evidence linking irAE to ICI efficacy. The largest meta-analysis to date, including 22,749 patients across multiple cancer types, confirmed that cirAE were associated with significantly improved OS (HR = 0.65; 95% CI: 0.58–0.73) and PFS (HR = 0.69; 95% CI: 0.61–0.78) (16). In NSCLC specifically, Grangeon et al. and Jurado et al. reported consistent OS benefits, though neither study applied time-dependent methods (10, 24) The key methodological distinction is the use of time-dependent Cox regression. The majority of prior studies treated irAE status as a fixed baseline covariate, which is known to introduce immortal time bias (13, 14). Shankar et al. found that multisystem irAE—but not individual organ-specific events—retained prognostic significance in time-dependent analyses (15), suggesting that some irAE subtypes are more susceptible to immortal time bias than others. In our analysis, cirAE retained an independent association with improved OS after time-dependent correction (HR = 0.52; P = 0.002). One plausible explanation for this robustness is the relative manageability of cutaneous toxicity compared with visceral irAE, which often permits continued ICI exposure—a possibility we examine in greater detail below.

The apparent discordance between the substantial OS benefit (adjusted HR = 0.52) and the borderline single-cutpoint PFS landmark result (P = 0.127) is clarified by the time-varying nature of the cirAE effect on PFS demonstrated in Section 3.7. The proportional-hazards assumption was satisfied for cirAE in the OS model (Schoenfeld P = 0.820) but violated in the PFS model (P = 0.013). The complementary time-varying coefficient analysis showed that cirAE was associated with a strong reduction in progression hazard early after exposure classification, with this protective effect attenuating progressively over follow-up while remaining directionally protective through approximately the first year. Both a time-invariant Cox model and a single-cutpoint landmark analysis necessarily summarize this non-proportional effect as an average across the entire observation window, diluting the early protective signal and producing borderline conclusions. For OS, the proportional-hazards assumption was satisfied; the cumulative benefit of early disease control progressively manifests over longer follow-up and is captured unambiguously by a single hazard ratio. The two endpoints are therefore consistent in direction once the temporal structure of the effect is accounted for; the apparent discordance reflects different temporal dynamics rather than opposing biology.

The grade-stratified analysis suggested a severity-associated trend in OS that, if confirmed, would have clinical relevance. Grade 3+ cirAE was associated with the largest point estimate of OS benefit (HR = 0.33; median OS 47.7 months), exceeding the estimate observed with grade 1–2 events (HR = 0.51; median OS 36.1 months), with a significant trend across grade categories (P for trend < 0.001). One plausible explanation for this gradient lies in a fundamental distinction between cutaneous and visceral irAE in terms of clinical manageability.

Grade 3+ hepatitis, pneumonitis, and myocarditis typically require high-dose systemic corticosteroids, additional immunosuppressive agents, and permanent ICI discontinuation (8, 17). The resulting loss of drug exposure negates the survival benefit of immune activation. Grade 3+ cirAE, by contrast, can often be managed with topical corticosteroids, oral antihistamines, and emollients; systemic immunosuppression is generally not required, and ICI therapy can in many cases be continued with appropriate dermatologic support. In our institution, multidisciplinary collaboration between oncology and dermatology services facilitated early recognition and active local management of cirAE, allowing most patients with grade 3+ cutaneous toxicity to remain on ICI therapy.

Our data provide direct empirical support for this approach. In the grade 3+ cirAE subgroup (excluding SJS/TEN), 95.2% of patients were able to continue ICI therapy, either without interruption (42.9%) or after a brief temporary hold with successful resumption (52.4%). Only one patient (4.8%) required permanent discontinuation. This contrasts with grade 3+ visceral irAE in the published literature, where permanent discontinuation rates typically exceed 40–60% (15, 17). The high ICI continuation rate was facilitated by adherence to established ASCO and ESMO management algorithms (17, 25): grade 3 cutaneous toxicities were managed primarily with topical high-potency corticosteroids and, when necessary, short courses of systemic corticosteroids at moderate doses (0.5–1.0 mg/kg/day prednisone equivalent, median duration 18 days). This regimen falls well below the threshold (≥10 mg/day for ≥30 days) that Arbour et al. identified as detrimental to ICI efficacy (26), suggesting that cirAE management in our cohort did not compromise antitumor immunity.

One interpretation consistent with these observations is that grade 3+ cirAE may reflect more pronounced T-cell-mediated immune activation while remaining locally controllable, such that strong antitumor immunity is preserved rather than extinguished by toxicity management. The point estimate of HR = 0.33 in this subgroup is consistent with this interpretation. We emphasize, however, that this hypothesis cannot be confirmed by the present observational data: the grade 3+ subgroup comprised only 21 patients with 8 OS events, yielding wide confidence intervals (95% CI 0.16–0.69) and limiting statistical precision. Although time-dependent Cox regression mitigates immortal time bias, it cannot fully eliminate survivor bias: patients who develop grade 3+ cirAE and remain on ICI therapy are necessarily those who tolerated the toxicity well enough to continue treatment, which may itself correlate with favorable performance status, less aggressive tumor biology, or other unmeasured factors. The severity-associated trend is therefore best regarded as exploratory and hypothesis-generating, providing a rationale for prospective validation rather than a basis for immediate practice change. If confirmed in adequately powered prospective cohorts, the clinical implication would be that grade 3+ cirAE should not automatically trigger ICI discontinuation but rather prompt multidisciplinary evaluation, with the goal of maintaining ICI therapy when clinically safe.

Several biological hypotheses have been proposed to explain why cirAE may serve as a prognostic signal in ICI-treated patients, although direct mechanistic evidence is not available in the present study. The shared-epitope hypothesis posits that T cells activated by ICI may recognize cross-reactive antigens expressed on both tumor cells and normal skin tissue, generating concurrent antitumor immunity and cutaneous autoimmunity; preliminary evidence supporting this concept has been reported in melanoma and NSCLC (27, 28). Consistent with the skin being a preferential site of T-cell-mediated on-target, off-tumor toxicity, a phase 1 trial of CD5-knockout anti-CD5 CAR-T cells reported on-target, off-tumor effects primarily affecting skin tissue alongside potent antitumor activity (29).The skin is the body’s largest immune organ, harboring a dense network of dendritic cells, Langerhans cells, and tissue-resident memory T cells (30), which has led some authors to suggest that cutaneous reactions may be among the earliest detectable manifestations of systemic immune activation during ICI therapy. These mechanistic frameworks remain hypothetical in our study: we did not perform T-cell receptor sequencing, single-cell transcriptomics, or other immune-profiling assays that would be required to directly support them. Confirmation of a shared-epitope basis for the cirAE–OS association, and of the proposed differential roles of immune activation in OS versus PFS endpoints, will require dedicated mechanistic studies in cohorts with paired tumor and skin tissue analyses.

Several clinical implications may be considered, with the caveat that they require prospective validation before broad adoption. First, the development of cirAE during ICI therapy may serve as an accessible, real-world signal that complements—rather than replaces—formal imaging response assessment. Second, the observed severity-associated trend, if confirmed, would argue against reflexive ICI discontinuation in patients who develop grade 3+ cutaneous toxicity; instead, multidisciplinary evaluation with active dermatologic management may be reasonable when systemic toxicity does not supervene. Third, patients who do not develop cirAE may warrant attentive efficacy monitoring, although the absence of cirAE alone is not a sufficient basis for early treatment modification given the substantial fraction of cirAE-negative patients who nonetheless derive benefit from ICI therapy. None of these implications should be interpreted as directly actionable until confirmed in prospective cohorts with standardized cirAE assessment.

Several limitations should be acknowledged. First, the retrospective single-center design limits generalizability; prospective multicenter validation is needed. Second, the primary cohort excluded 36 patients with concurrent non-cutaneous irAEs to isolate cirAE-specific effects. Sensitivity analysis re-including these patients yielded consistent results, but a future study designed to model cirAE-by-concurrent-irAE interactions would more directly address effect modification. Third, the grade 3+ cirAE subgroup was small (n=21, 8 events), yielding wide confidence intervals. Time-dependent Cox regression mitigates immortal time bias but cannot eliminate the survivor bias inherent in observational data. The severity-associated trend is therefore exploratory. Fourth, treatment-exposure metrics (ICI cycles, treatment duration) were not modeled as covariates because they are determined by survival itself and lie on the causal pathway. Consequently, part of the observed association may reflect treatment-exposure dynamics that cannot be cleanly separated from immune biology. Finally, detailed data on post-progression treatment regimens and duration were unavailable, precluding adjustment for subsequent therapies that could influence overall survival. Additionally, comprehensive molecular biomarkers (e.g., tumor mutation burden) were unavailable for many patients, and mechanistic validation via immune profiling was not performed.

## 5 Conclusion

cirAE independently associated improved OS in ICI-treated advanced NSCLC, a finding that withstood correction for immortal time bias through both time-dependent Cox regression and was supported by a complementary landmark visualization. The proportional-hazards assumption was formally tested: the cirAE–OS association was time-invariant, whereas the cirAE–PFS relationship was time-varying, with the early protective effect attenuating over follow-up. Once this temporal structure is accounted for, the OS and PFS findings are consistent in direction. A grade-dependent association was observed, with higher-grade cirAE showing larger point estimates of OS benefit. This exploratory severity-associated trend requires prospective validation before informing clinical practice. cirAE—particularly higher-grade events—may be recognized as a candidate prognostic signal rather than an indication for automatic treatment discontinuation in this cohort, with the recognition that whether dermatologic management to maintain ICI therapy through grade 3+ cutaneous toxicity is causally beneficial cannot be determined from observational data and requires prospective validation. Prospective multicenter studies with standardized irAE assessment and molecular profiling will be needed to confirm these associations and identify the immune pathways that connect skin toxicity to antitumor efficacy.

## Data Availability

The raw data supporting the conclusions of this article will be made available by the authors, without undue reservation.
