# Supplementary material for: Cutaneous immune-related adverse events independently predict overall survival with a grade-dependent association in ICI-treated advanced NSCLC: a time-dependent analysis
Source: Front Oncol. 2026 Aug 12;16:1860717. doi: 10.3389/fonc.2026.1860717 (PMC13462687; doi:10.3389/fonc.2026.1860717)
Supplement: Supplementary file 1 [file Table1.docx]

| **Variable** | **Univariable HR (95% CI)** | **P value** | **Multivariable HR (95% CI)** | **P value** |
| --- | --- | --- | --- | --- |
| cirAE† | 0.52 (0.38 to 0.72) | <0.001 | 0.56 (0.39 to 0.81) | 0.002 |
| Concurrent non-cutaneous irAE |  |  |  |  |
| No | 1 [Reference] |  | 1 [Reference] |  |
| Yes | 0.70 (0.44 to 1.11) | 0.128 | 0.87 (0.50 to 1.52) | 0.618 |
| Sex |  |  |  |  |
| Female | 1 [Reference] |  | 1 [Reference] |  |
| Male | 0.84 (0.59 to 1.21) | 0.354 | 0.93 (0.63 to 1.36) | 0.696 |
| Age, years |  |  |  |  |
| <65 | 1 [Reference] |  | 1 [Reference] |  |
| ≥ 65 | 0.92 (0.69 to 1.24) | 0.605 | 0.79 (0.58 to 1.08) | 0.141 |
| ECOG PS |  |  |  |  |
| 0-1 | 1 [Reference] |  | 1 [Reference] |  |
| 2 | 0.81 (0.49 to 1.33) | 0.404 | 1.17 (0.64 to 2.15) | 0.606 |
| Clinical stage |  |  |  |  |
| III | 1 [Reference] |  | 1 [Reference] |  |
| IV | 2.73 (1.83 to 4.08) | <0.001 | 2.91 (1.90 to 4.46) | <0.001 |
| Histology |  |  |  |  |
| Non-squamous | 1 [Reference] |  | 1 [Reference] |  |
| Squamous | 1.04 (0.78 to 1.38) | 0.775 | 1.43 (1.05 to 1.96) | 0.025 |
| PD-L1 TPS |  |  |  |  |
| <1% | 1 [Reference] |  | 1 [Reference] |  |
| 1-49% | 0.55 (0.38 to 0.80) | 0.002 | 0.46 (0.31 to 0.68) | <0.001 |
| ≥ 50% | 0.58 (0.36 to 0.93) | 0.025 | 0.38 (0.23 to 0.63) | <0.001 |
| Unknown | 1.04 (0.72 to 1.51) | 0.833 | 0.97 (0.66 to 1.42) | 0.877 |
| Treatment regimen |  |  |  |  |
| Chemotherapy + ICI | 1 [Reference] |  | 1 [Reference] |  |
| ICI monotherapy | 1.40 (0.96 to 2.04) | 0.077 | 1.47 (0.80 to 2.69) | 0.212 |
| Line of therapy |  |  |  |  |
| First-line | 1 [Reference] |  | 1 [Reference] |  |
| Second-line | 1.11 (0.79 to 1.57) | 0.533 | 0.77 (0.45 to 1.31) | 0.332 |

Supplementary Table S1a. Univariable and Multivariable Time-Dependent Cox Regression for Overall Survival in the Expanded Cohort (n=298)

Abbreviations:Any cirAE includes isolated cirAE (n=80) and cirAE concurrent with non-cutaneous irAE (n=36). Reference group: no irAE (n=182). Multivariable model adjusted for all listed variables.
