# Supplementary material for: Cutaneous immune-related adverse events independently predict overall survival with a grade-dependent association in ICI-treated advanced NSCLC: a time-dependent analysis
Source: Front Oncol. 2026 Aug 12;16:1860717. doi: 10.3389/fonc.2026.1860717 (PMC13462687; doi:10.3389/fonc.2026.1860717)
Supplement: Supplementary file 2 [file Table2.docx]

| **Variable** | **Univariable HR (95% CI)** | **P value** | **Multivariable HR (95% CI)** | **P value** |
| --- | --- | --- | --- | --- |
| cirAE† | 0.79 (0.60 to 1.04) | 0.088 | 1.00 (0.73 to 1.37) | 0.989 |
| Concurrent non-cutaneous irAE |  |  |  |  |
| No | 1 [Reference] |  | 1 [Reference] |  |
| Yes | 0.73 (0.48 to 1.11) | 0.143 | 0.68 (0.40 to 1.16) | 0.156 |
| Sex |  |  |  |  |
| Female | 1 [Reference] |  | 1 [Reference] |  |
| Male | 0.73 (0.53 to 0.99) | 0.041 | 0.77 (0.56 to 1.08) | 0.129 |
| Age, years |  |  |  |  |
| <65 | 1 [Reference] |  | 1 [Reference] |  |
| ≥65 | 0.95 (0.73 to 1.23) | 0.686 | 0.88 (0.67 to 1.16) | 0.373 |
| ECOG PS |  |  |  |  |
| 0-1 | 1 [Reference] |  | 1 [Reference] |  |
| 2 | 0.82 (0.52 to 1.29) | 0.389 | 1.15 (0.63 to 2.09) | 0.653 |
| Clinical stage |  |  |  |  |
| III | 1 [Reference] |  | 1 [Reference] |  |
| IV | 2.15 (1.55 to 2.97) | <0.001 | 2.52 (1.76 to 3.59) | <0.001 |
| Histology |  |  |  |  |
| Non-squamous | 1 [Reference] |  | 1 [Reference] |  |
| Squamous | 1.05 (0.81 to 1.35) | 0.728 | 1.49 (1.12 to 1.97) | 0.005 |
| PD-L1 TPS |  |  |  |  |
| <1% | 1 [Reference] |  | 1 [Reference] |  |
| 1-49% | 0.62 (0.45 to 0.86) | 0.005 | 0.60 (0.43 to 0.85) | 0.004 |
| ≥50% | 0.61 (0.40 to 0.94) | 0.024 | 0.47 (0.29 to 0.74) | 0.001 |
| Unknown | 0.84 (0.60 to 1.18) | 0.321 | 0.83 (0.59 to 1.18) | 0.306 |
| Treatment regimen |  |  |  |  |
| Chemotherapy + ICI | 1 [Reference] |  | 1 [Reference] |  |
| ICI monotherapy | 1.50 (1.08 to 2.08) | 0.016 | 1.63 (1.02 to 2.62) | 0.041 |
| Line of therapy |  |  |  |  |
| First-line | 1 [Reference] |  | 1 [Reference] |  |
| Second-line | 1.22 (0.91 to 1.64) | 0.186 | 0.78 (0.51 to 1.21) | 0.265 |

Supplementary TableS1b. Univariable and Multivariable Time-Dependent Cox Regression for Progression-Free Survival in the Expanded Cohort (n=298)

Abbreviation:Any cirAE includes isolated cirAE (n=80) and cirAE concurrent with non-cutaneous irAE (n=36). Reference group: no irAE (n=182). Three patients with PFS events before cirAE onset were classified as unexposed. Multivariable model adjusted for all listed variables.
